# A Systematic Proteomic Study of Irradiated DNA Repair Deficient *Nbn*-Mice

**DOI:** 10.1371/journal.pone.0005423

**Published:** 2009-05-01

**Authors:** Anna Melchers, Lars Stöckl, Janina Radszewski, Marco Anders, Harald Krenzlin, Candy Kalischke, Regina Scholz, Andreas Jordan, Grit Nebrich, Joachim Klose, Karl Sperling, Martin Digweed, Ilja Demuth

**Affiliations:** 1 Institut für Humangenetik, Center for Biomedical Nanotechnology, Charité - Universitätsmedizin Berlin, Campus Virchow Klinikum, Berlin, Germany; 2 Medizinische Klinik mit Schwerpunkt Hämatologie/Onkologie, Center for Biomedical Nanotechnology, Charité - Universitätsmedizin Berlin, Campus Virchow Klinikum, Berlin, Germany; 3 Klinik für Strahlenheilkunde, Center for Biomedical Nanotechnology, Charité - Universitätsmedizin Berlin, Campus Virchow Klinikum, Berlin, Germany; Buck Institute for Age Research, United States of America

## Abstract

**Background:**

The *NBN* gene codes for the protein nibrin, which is involved in the detection and repair of DNA double strand breaks (DSBs). The *NBN* gene is essential in mammals.

**Methodology/Principal Findings:**

We have used a conditional null mutant mouse model in a proteomics approach to identify proteins with modified expression levels after 4 Gy ionizing irradiation in the absence of nibrin *in vivo*. Altogether, amongst ∼8,000 resolved proteins, 209 were differentially expressed in homozygous null mutant mice in comparison to control animals. One group of proteins significantly altered in null mutant mice were those involved in oxidative stress and cellular redox homeostasis (p<0.0001). In substantiation of this finding, analysis of *Nbn* null mutant fibroblasts indicated an increased production of reactive oxygen species following induction of DSBs.

**Conclusions/Significance:**

In humans, biallelic hypomorphic mutations in *NBN* lead to Nijmegen breakage syndrome (NBS), an autosomal recessive genetic disease characterised by extreme radiosensitivity coupled with growth retardation, immunoinsufficiency and a very high risk of malignancy. This particularly high cancer risk in NBS may be attributable to the compound effect of a DSB repair defect and oxidative stress.

## Introduction

The most dangerous DNA lesion caused by ionizing irradiation (IR) is considered to be the double-strand break (DSB). Evolution has furnished the mammalian cell with several mechanisms for responding to the induction of this critical DNA lesion. A central and essential component of the DNA damage response is nibrin, the product of the *NBN* gene [1], [2]. Nibrin forms a trimeric complex with Mre11 and Rad50 (MRN complex), which is conserved between yeast and mammals. This complex localizes to the sites of DSBs where the DNA binding function of RAD50 and the nucleolytic activity of MRE11 contribute to DNA repair, by both homologous recombination and non homologous end-joining. In addition, the MRN complex at the sites of DSBs promotes the activation of the ATM kinase which is mutated in the genetic disease Ataxia-telangiectasia (A-T) [3], [4]. Downstream targets of ATM are then responsible for cell cycle arrest at the major checkpoints. Since nibrin is required for the relocalisation of the MRN complex to DSBs it occupies a critical position in the DNA damage response cascade.

Not surprisingly, therefore, *NBN* is an essential gene, however, individuals with hypomorphic mutations in the *NBN* gene [5] suffer from the autosomal recessive genetic disorder, Nijmegen Breakage Syndrome (NBS, MIM 251260). Since null mutation of the *Nbn* gene is lethal in the mouse [6], [7], we have previously used Cre recombinase/loxP technology to generate mice with conditional null mutation in the *Nbn* gene [8], [9]. Induction of *Nbn* null mutation *in vivo* in this conditional mouse model results in chromosome damage, radiomimetic-sensitivity, cell cycle checkpoint defects and impaired immunoglobulin class switching. Homozygous null mutant cells survive only briefly in culture and in proliferating tissues, such as bone marrow, and are rapidly replaced by heterozygous null mutant cells. However, in liver tissue, null mutant cells survived well and even after two weeks still 95% of this organ consisted of null mutant cells [8].

The liver thus provides an excellent *in vivo* model to investigate the consequences of a complete lack of nibrin during a DNA damage response. Here we have used a proteomics approach to identify proteins which are differentially expressed in response to IR in liver tissue homozygous for the *Nbn* null mutation. This analysis revealed an altered expression pattern of various proteins involved in the cellular response to oxidative stress and strongly suggests a link between DSB repair and the generation of reactive oxygen species (ROS) during the DNA damage response. These findings indicate an unexpected role for ROS-detoxification in the pathophysiology of NBS.

## Results and Discussion

### *In vivo* induction of *Nbn* mutations by Cre recombinase

All the mice used in this study had one *Nbn* allele in which exon 6 was flanked by *loxP* sites, *Nbn* ^lox-6^. The other allele was either wild type or carried a further *Nbn* null mutation, *Nbn* ^ins-6^. All mice were also transgenic for Cre recombinase under the control of the interferon responsive promoter, Mx1 [10]. This allowed us to induce *in vivo* the conversion of the *Nbn* ^lox-6^ allele to the *Nbn* ^del-6^ allele by injection of the mice with poly(I):poly(C) resulting in induction of interferon, Cre recombinase expression and exon 6 deletion.

**Figure 1:**
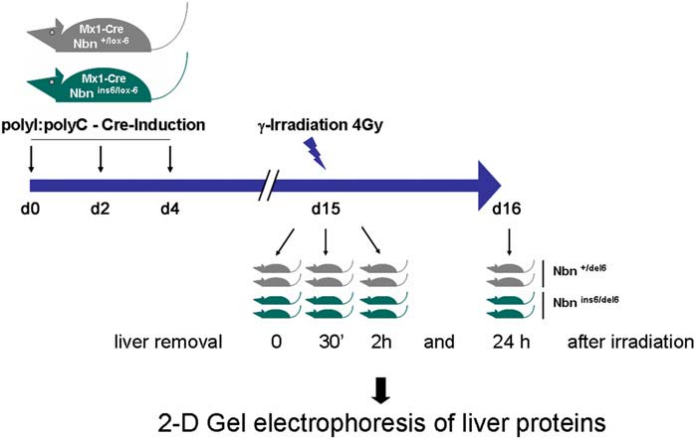
Experimental workflow of the proteomic study on *Nbn* null mutant mouse livers following IR. Eight mice of each genotype, either *Nbn* ^+/lox-6^ or *Nbn* ^ins-6/lox-6^, were injected three times with poly(I):poly(C) in order to achieve cre recombinase expression in interferon sensitive tissue and, as a result, deletion of the lox-6 allele in liver cells. The mice were irradiated (4 Gy) 15 days after the first poly(I):poly(C) injection and the livers were removed 0, 0.5 h, 2 h and 24 h after irradiation for subsequent proteomic analysis.

Altogether eight mice of each genotype (*Nbn* ^+/del-6^ and *Nbn* ^ins-6/del-6^) were compared in this proteomic study. Two mice were killed 0, 0.5, 2 and 24 hours after 4 Gy irradiation. Total proteins and DNA were extracted from liver tissue. The DNA was used in a semi-quantitative PCR assay in order to determine the efficiency of cre recombinase mediated deletion of *Nbn* exon 6 and thus the proportion of homozygous or heterozygous null mutant cells. As shown in Table 1 this efficiency was in all cases clearly above 90%, allowing the analysis of liver tissue essentially in the absence of nibrin. Supplementary information on the cre recombinase mediated deletion efficiency assayed at the protein level is available as Supplementary Figure S1. An overview of the experimental approach used here for for the proteome analysis of *Nbn* null mutant mice following IR is shown in Figure 1.

**Table 1:** Efficiency of the *in vivo* deletion of *Nbn* exon 6 by Cre recombinase in mouse liver tissue.

Initial *Nbn* genotype	Irradiation (Gy)/time point of 2-DE analysis (h)	Deletion efficiency in %, mean of the two mice used for 2-DE
ins-6/lox-6	0	94.04 (±3.56)
ins-6/lox-6	4/0.5	93.11 (±1.71)
ins-6/lox-6	4/2	97.65 (±2.35)
ins-6/lox-6	4/24	95.06 (±0.64)
+/lox-6	0	94.53 (±1.94)
+/lox-6	4/0.5	96.96 (±3.05)
+/lox-6	4/2	94.86 (±1.51)
+/lox-6	4/24	96.50 (±3.50)

### Liver proteome analysis of irradiated *Nbn* ^+/del-6^ and *Nbn* ^ins-6/del-6^ mice by 2-DE

**Figure 2:**
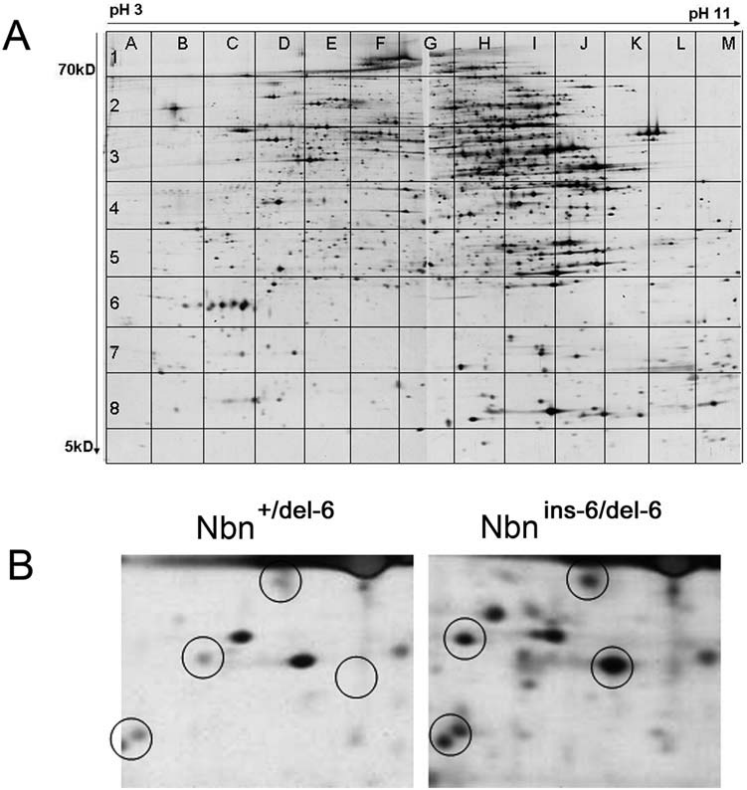
Overview of liver proteins differentially expressed in mice heterozygote or homozygote for *Nbn* null mutations following IR. A) A representative high resolution 2-DE liver protein pattern from a non irradiated mouse is shown with a resolution of approximately 8000 discrete protein spots. Proteins were visualized by silver staining. Protein spots which differ in intensity in the patterns from mice heterozygous or homozygous for *Nbn*-null mutations were identified and mapped using the grid system shown. B) A 2-DE gel section from coordinates D3 (Figure 1A) from liver samples of *Nbn* ^+/del-6^ and *Nbn* ^ins-6/del-6^ mice 24 h after irradiation with 4 Gy is shown as an example of protein spot alterations. Several differences in protein spot intensity are visible (circled).

Total Proteins were separated by two-dimensional gel electrophoresis. After silver staining of the 2-DE gels, we detected approximately 8000 discrete spots per sample (Figure 2A). Comparisons were made between gels of homozygous and heterozygous mutated animals. This allowed us to exclude non-specific protein changes due solely to the injection of poly(I):poly(C) or the expression of cre recombinase. An example of equivalent gel sections from samples of heterozygous and homozygous mutated animals with several spots displaying differences in abundance is given in Figure 2B. For analyses, only those protein spots which were changed in intensity in both mice were considered to be significantly altered.

**Figure 3:**
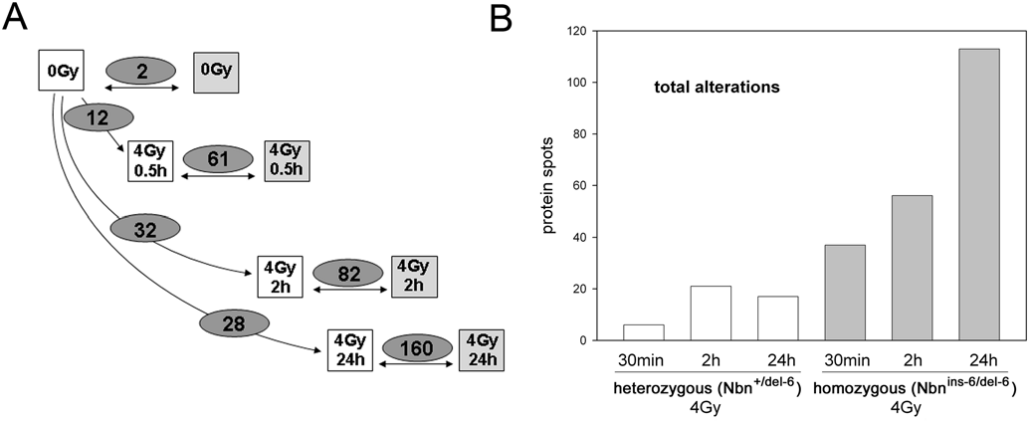
The experimental setup and protein spot alterations in livers from *Nbn* ^+/del-6^ and *Nbn* ^ins-6/del-6^ mice at different time points after irradiation with 4 Gy: All proteins. A) The experimental setup: Spots with altered protein abundance were identified by pairwise visual comparison of gels from heterozygous and homozygous *Nbn*-null mutant mice; *Nbn* ^+/del-6^ mice in white boxes, *Nbn* ^ins-6/del-6^ mice in grey boxes. The numbers in the ovals are the number of altered spots for the time points given after 4 Gy irradiation. B) The bar chart shows proteins with altered expression levels in the livers of heterozygous and homozygous *Nbn*-null mutant mice after irradiation as identified by mass spectrometry. *Nbn* ^+/del-6^ mice in white bars, *Nbn* ^ins-6/del-6^ mice in grey bars.

Prior to irradiation there were essentially no differences between heterozygous and homozygous mice. Only two proteins, glutathione synthetase and serine (or cysteine) proteinase inhibitor, were found to be upregulated in *Nbn* ^ins-6/del-6^ mice. After irradiation, heterozygous mice showed altered expression of several proteins with a maximum of 32 altered protein spots 2 hours after irradiation (Figure 3A). Homozygous animals showed at each time point much greater effects on protein composition. Unlike heterozygous animals there were a maximum number of altered protein spots at 24 hours post IR: 160 altered spots in comparison to heterozygous control animals (Figure 3A).

### Changes in proteins associated with oxidative stress, metabolism and heat shock response/chaperoning

**Figure 4:**
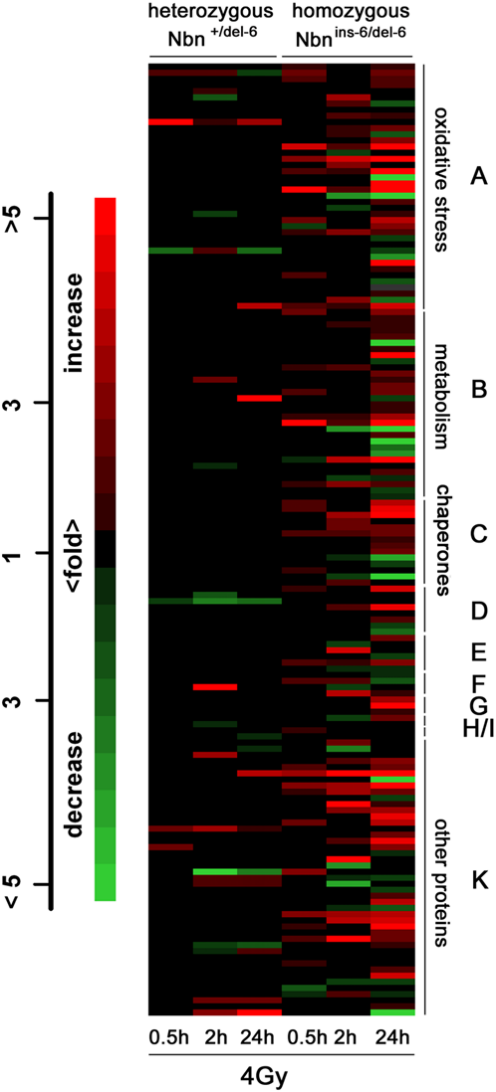
Radiation-induced protein alterations in livers from *Nbn* ^+/del-6^ and *Nbn* ^ins-6/del-6^ mice. The relative amount of protein in altered spots was measured densitometrically and their identity was determined by mass spectrometry. In the heat plot, the measured abundance of individual proteins was converted to the colour scale given on the left. Identified proteins were assigned to ontological groups by the ProfCom program and these are given on the right hand side: A, Proteins involved in oxidative stress (oxidoreductase activity, cell redox homeostasis); B, Proteins involved in metabolism (metabolic process); C, Chaperones and heat shock proteins (unfolded protein binding); D, Proteins involved in cell cycle regulation; E, Proteasomal proteins; F, Proteins involved in nucleic acids metabolism; G, Proteins involved in gene expression; H, Proteins involved in apoptosis; I, Protease inhibitors; K, other proteins.

Protein spots, which were initially identified visually as altered in abundance between the compared gels, were further evaluated quantitatively using the Proteomeweaver 2.1 imaging software as described in the methods section. Quantitatively altered spots were excised from gels and subjected to mass spectrometry (MS) for protein identification. We were able to identify a total of 147 protein spots (Figure 3B) and have assigned them to ontological groups using the web based database ProfCom (Supplementary Table S1). The heat plot in Figure 4 shows the fluctuations in abundance of these 147 proteins for mice of both genotypes. Animals heterozygous for the *Nbn* null mutation showed transient changes in the expression of proteins involved in the cellular response to oxidative stress, in metabolism and in the chaperone/heat shock proteins (Supplementary Figure S2).

Disturbances in proteins involved in the cellular response to ROS have been reported previously in a proteomic analysis of the livers of irradiated wild type rats [11]. Homozygous *Nbn* null mutant mice, however, showed a much greater and prolonged perturbation in the expression of these proteins. For example, Peroxiredoxin 6 is an important antioxidant enzyme [12] known to be upregulated at the mRNA level in response to oxidative stress [13], which we have found at elevated levels 0.5 h and 2 h post IR in heterozygous animals. In homozygous null mutants this protein is still upregulated 24 h after IR (Supplementary Table S1). Manganese superoxide dismutase (SOD2) is another example from the group of proteins upregulated in homozygous mice even 24 h post IR. The enrichment of the identified proteins with oxidoreductase activity or involvement in cell redox homeostasis as determined using the ProfCom database was statistically highly significant (p<0.0001). Whilst the majority of the identified proteins are upregulated in response to IR, some are also down-regulated such as the aldehyde dehydrogenases 7 and 9 (Figure 4 and Supplementary Table S1).

Proteins of the metabolic process group with altered protein expression are involved in the citric acid cycle, gluconeogenesis, glycolysis and other important pathways. The heat shock protein HSP60 was found in as many as six protein spots with a greatly increased protein level in mice homozygous for the null mutation (Supplementary Table S1). The specific effects on the proteome observed here can be attributed to the basic defect in NBS, defective DNA repair [14].

### The DNA repair defect and elevated ROS in NBS

NBS is a chromosome instability syndrome but although the MRN complex is clearly involved in the repair of DSBs, biochemical measurements of DSB repair in NBS patient cells have consistently indicated that it follows normal kinetics [15], [16]. This clearly contrasts to the high levels of spontaneous and IR-induced chromosomal aberrations in NBS patient cells. This disparity may reflect compromised fidelity of DSB repair in NBS cells rather than reduced efficacy. Murine fibroblasts with the same null mutations employed here have been reported to show reduced DSB repair by both homology-directed gene conversion and SSA with an accompanying increase in repair by error-prone NHEJ [17]. Such null mutant cells show even higher levels of chromosome damage than patient cells [8], [17] reflecting rescue by the hypomorphic p70-nibrin protein present in patient cells.

A careful analysis using constant field gel electrophoresis has indicated that a major difference between NBS and control cells is in the number of residual unrepaired DSBs remaining 24 hours after irradiation: 3.3%±0.9% in normal cells, 5.6% in NBS cells [18]. Residual DSBs at 24 hours correlate with cell survival in mammalian cells [18], [19]. In addition to DSB-based gene mutations, which other factors contribute to reduced survival in irradiated NBS cells? The data presented here suggest that a key consequence of unrepaired DSBs in NBS cells may be disturbances in cell redox homeostasis.

**Figure 5:**
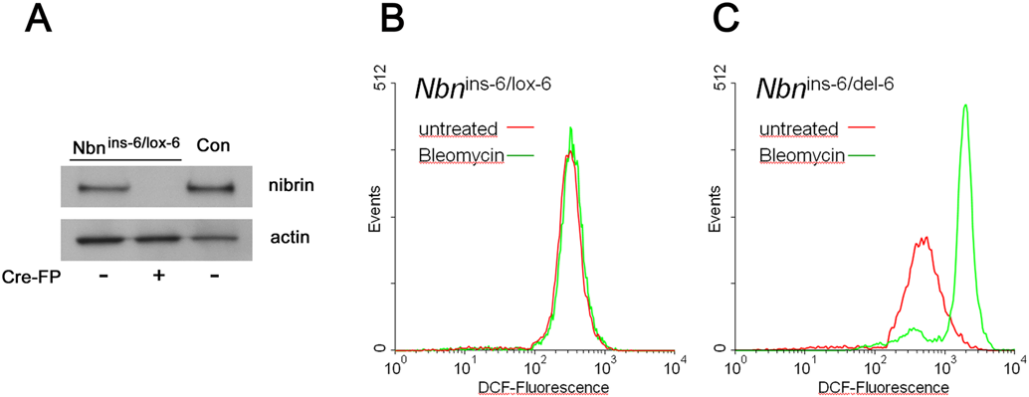
Elevated ROS levels due to DSBs in *Nbn* null mutant fibroblasts. A) Immunoblot for nibrin in *Nbn* ^ins-6/lox-6^ fibroblast lysates with and without treatment with a cre recombinase fusion protein (Cre-FP). Con: lysates from wild type mouse cells. B) FACS analysis of *Nbn* ^ins-6/lox-6^ fibroblasts incubated for 12 hours in Bleomycin and then labelled with CM-H_2_DCFDA. Conversion of CM-H_2_DCFDA to the flourescent derivative DCF reflects the ROS content of the analyzed cells. C) As in B except that the cells were treated 48 hours previously with Cre recombinase to yield null mutant *Nbn* ^ins-6/del-6^ fibroblasts. The experiment was repeated with independently prepared cells and gave essentially the same results.

Free radicals are, on the one hand, mutagenic and, on the other, result in general cell damage, apoptosis and necrosis [20], [21]. The upregulation of genes involved in detoxification of ROS is the natural consequence. The cell has three main defence mechanisms for dealing with ROS. Firstly, these radicals can be inactivated by direct interaction with low molecular weight antioxidants or scavengers such as vitamin E, NADH and glutathione. A second line of defence employs antioxidant enzymes such as the superoxide dismutases (SOD) and glutathione-S-transferases to detoxify ROS. Finally, damaged macromolecules can be directly repaired by enzymes such as disulfide reductase and methionine sulfoxide reductase. It is just such proteins which were found here to be increased in the livers of irradiated null mutant mice. It is perhaps noteworthy, that of the two proteins differing between unirradiated heterozygous and homozygous null-mutant mice one was gutathione-synthetase. Finally, the alterations in the chaperone and heat shock protein group as detected here also indicate elevated cellular ROS levels [22]. The persistent upregulation of ROS related proteins in the livers of irradiated null mutant mice suggests that ROS generation continues after the initial DNA damaging event. In contrast, in control animals with sufficient nibrin, DNA repair processes avert further ROS production. In order to test this hypothesis we turned to dermal fibroblasts previously isolated from these conditional knock out mice [2], [8]. As shown in Figure 5A, deletion of the *loxP*-flanked exon 6 in these *Nbn* ^ins-6/lox-6^ cells can be achieved by incubation with a Cre recombinase fusion protein [23].

For induction of DSBs, we used the radiomimetic, Bleomycin. Bleomycin intercalates into DNA and, in a two step, oxygen and metal ion dependent reaction, cleaves both DNA strands [24]. The ratio of DSBs to single-strand breaks after treatment with Bleomycin is 1∶9, in comparison, the ratio for IR is 1∶100 [25]. Bleomycin is thus particularly well suited to test our hypothesis. The Bleomycin concentration used here is approximately equivalent to 2 Gy IR [26] leading to on the order of 60 DSBs/cell [27]. For quantification of ROS, cells were stained with CM-H_2_DCFDA which is converted to the fluorescent compound, dichlorofluorescein (DCF) by reaction with hydrogen peroxide, hydroxyl radicals or peroxyl radicals [28], [29].

As shown in the FACS analysis in Figure 5B, after 12 hours incubation in Bleomycin, *Nbn* ^ins-6/lox-6^ cells show no increase in DCF-fluorescence in comparison to untreated cells. However, if the cells are converted by Cre recombinase to null mutant *Nbn* ^ins-6/del-6^ cells, Bleomycin treatment leads to a four-fold increase in intracellular ROS levels (Figure 5C). A similar level of DCF-fluorescence was obtained when *Nbn* ^ins-6/lox-6^ cells were incubated for 12 hours in hydrogen peroxide (data not shown). Clearly the DNA repair defect in *Nbn* null mutants leads to increased ROS production and, in consequence, the specific effects on the proteome reported here.

### Redox homeostasis and NAD^+^ levels in irradiated *Nbn* null mutant mice

**Figure 6:**
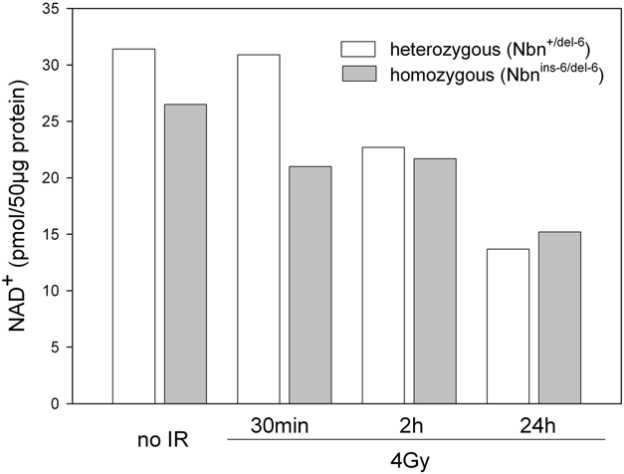
NAD^+^ levels in the mouse liver tissues used for the proteomic analysis. The NAD^+^ level of liver tissue from all 16 animals analysed in the proteomic study was determined. Each bar represents the mean value of the two mice with identical genotype (*Nbn* ^+/del-6^ or *Nbn* ^ins-6/del-6^) and treatment (IR).

One link between redox homeostasis and unrepaired DSBs could be NAD depletion by poly (ADP-ribose) polymerase (PARP). Inactive chromatin-bound PARP is activated by DNA strand breaks and cleaves NAD to yield nicotinamide and ADP-ribose molecules which are then added as polymers to many nuclear proteins. Given that PARP activation is proportional to the number of DSBs [30], higher levels of residual DSBs must lead to increased PARP-directed cleavage of NAD. Since NAD(P)H is a directly acting antioxidant, depletion of NAD would have the exacerbating effect of actually increasing free radical levels [31], [32]. In order to test this hypothesis we determined the NAD^+^ content from liver tissue of all 16 animals analyzed in the proteomic study (Figure 6). Depletion of NAD^+^ was clearly observed after irradiation of mice with either genotype. At 30 minutes after irradiation, when 60% of DSBs will normally have been repaired, livers from null mutant mice were more strongly depleted for NAD^+^. This could well reflect hyperactivation of PARP due to unrepaired DSBs. At the later time points after irradiation, NAD^+^ levels were indistinguishable between the control and null mutant mice, reflecting the effective restoration of redox homeostasis after upregulation of the genes detected here.

### Reactive oxygen species and the clinical features of NBS

Cancer occurrence in NBS is the highest amongst the chromosome instability disorders and this may reflect the combined mutagenic burden of DSBs and free radicals. Similarly, growth retardation in NBS, which has generally been considered to reflect retarded cell proliferation and senescence may also, at least in part, be due to ROS-triggered apoptosis and necrosis [21], [33]. In this respect it is interesting that neurons, with their extreme metabolic activity already generating large amounts of ROS, are particularly vulnerable to oxidative stress [20], [21]. Thus, the microcephaly characteristic for NBS patients may reflect this tissue specific sensitivity. As we have previously shown, mice with the *Nbn* null mutations used here but targeted exclusively to neurons have a severe neurological phenotype with extreme cerebellar disruption and ataxia [34]. The fact that NBS patients do not have these symptoms can now be attributed to p70-nibrin's (partial) rescue of both DSB repair and ROS detoxification.

NBS is a disorder affecting particularly B-lymphocytes. Most patients have reduced IgA and/or IgG levels with essentially unaffected IgM. However, the immunodeficiency in NBS patients is not purely humoral there is also a clear defect in cellular immunity. B-lymphocytes are exposed to considerable redox fluctuations during activation and inflammation, thus the balance of antioxidant systems, on the one hand, and ROS-mediated signal transduction, on the other, is critical. Indeed, exogenous ROS have been shown to have a suppressive effect on B-lymphocyte activation [35] and this could be of relevance for NBS if levels of ROS are elevated due to their DSB repair defect.

In summary, key clinical characteristics of NBS can now be understood as the consequences of a combination of DSB repair defect and associated overproduction of ROS due to accelerated depletion of NADH and other cellular antioxidants. Similar conclusions have been drawn for the related disease, A-T [36], where reduced levels of antioxidant activity and increased sensitivity to oxidative stress have been directly demonstrated [37], [38]. Nibrin is essential for regular autophosphorylation and activation of ATM in response to DSBs [3], [4], [39]. Is the disturbed redox homeostasis observed here in null mutant mouse livers simply a consequence of the inability to activate ATM in the absence of nibrin? The lethality of disruption of the MRN genes in mice compared to the viability of ATM null mutant animals indicates a clear difference in the nature of the underlying genes. In contrast to the signal transducer ATM, the MRN complex has explicit functions involving DSB-binding and enzymatic activities during DNA repair [40], [41]. Thus DSBs are likely to have an even greater impact in NBS cells than in A-T cells independently of the activation of ATM. The greatly disturbed redox homeostasis described here for *Nbn* null mutants further highlights the functional distinction between ATM and nibrin.

Clinical variability in NBS is quite considerable. We have recently demonstrated that the amount of p70-nibrin expressed correlates with cancer incidence [42]. The results presented here suggest that individual variation in genes involved in response to oxidative stress may have a similar impact on disease progression. This hypothesis can now be addressed epidemiologically in patient cohorts and experimentally in mouse models.

## Methods

### Mouse breeding, *in vivo* induction of *Nbn* null mutations and irradiation

To induce *Nbn* null mutations *in vivo*, we crossed mice to generate animals which express the Cre recombinase under control of the interferon-responsive promoter, Mx1 [10] and carrying one allele with *Nbn* exon 6 flanked by LoxP sites [8] and the other allele being either wildtype or a null mutant allel with an insertion in *Nbn* exon 6 [6]. Mice were identified by PCR genotyping of DNA isolated from tail biopsies. Cre recombinase mediated induction of *Nbn* null mutations *in vivo* was done as previously described [8]. Briefly, Cre expression was induced by intraperitoneal injection of the interferon inducer, poly(I):poly(C) (Amersham-Pharmacia). Each mouse received three 250 µl injections of a 2 mg/ml solution of poly(I):poly(C) in water every 48 h. Mice were irradiated using the X-ray apparatus Gulmay Medical D3-225 at a dose rate of 0.77 Gy/min 15 days after the first injection and analyzed at the indicated time points post IR. Livers were removed from mice after cervical dislocation and portions processed for DNA and Protein extraction. Animal maintenance and experimental procedures were in accordance with the German Animal Welfare Act and were approved by the Landesamt für Arbeitsschutz, Gesundheitsschutz und technische Sicherheit Berlin (G 0084/02).

### Semi-quantitative PCR and immunoblotting for the estimation of Cre recombinase-mediated *Nbn* exon 6 deletion

The semi-quantitative PCR strategy used to verify the *in vivo* deletion of the *Nbn* exon 6 induced by Cre recombinase expression was described previously in detail [8]. In brief, similarly sized PCR products were amplified from the three alleles: *Nbn* ^+^, *Nbn* ^del-6^ and *Nbn* ^lox-6^ with one oligonucleotide carrying a fluorescein label. Undenatured PCR products were separated on native 12% polyacrylamide gels, which were then analysed in the ‘vistra FluorImager SI’ (Amersham Life Science) using bandpass emission filter 530 DF 30 and ImageQuaNT software (Molecular Dynamics, version 5.2). All semi-quantitative PCRs were conducted with 27 cycles. The intensity of the fluorescence signals was used to calculate the proportion of the alleles and thus the proportion of homozygous or heterozygous null mutant cells in the sample. Protein lysates from aliquots of cells prepared for ROS measurement were analysed for *Nbn* exon6 deletion efficiency by immunoblotting using standard methods and as described before [43]. Immunoblots were probed with antibodies recognising mouse nibrin [8] and actin (abcam).

### Protein extraction procedure

Total protein extracts were prepared from perfused frozen livers by an extraction procedure described previously, with some modifications [44], [45]. In short, frozen liver tissue, 1.6 parts v/w of buffer 1 (0.11 M CHAPS, 50 mM TRIZMA® Base (Sigma-Aldrich, Steinheim, Germany), 50 mM KCl and 20% w/v glycerol at pH 7.5), 0.08 parts of protease inhibitor solution I (1 Complete™ tablet (Roche Applied Science, Mannheim, Germany) dissolved in 2 ml of buffer 1) and 0.02 parts of protease inhibitor solution II (1.4 µM pepstatin A and 1 mM phenylmethylsulfonyl fluoride in ethanol) were ground to fine powder in a mortar precooled in liquid nitrogen. The tissue powder was transferred into a 2 ml tube, quickly thawed and an average number of 11 glass beads added (0.034 units of glass beads per combined weight of tissue, buffers and inhibitors in mg). Each sample was sonicated 6 times in an ice-cold water bath for 15 s each, with cooling intervals of 1 min 45 s in between. The homogenate was stirred for 30 min in the presence of 0.025 parts v/w of benzonase (Merck, Darmstadt, Germany) and 0.021 parts v/w 5 mM magnesium chloride in buffer 1 without CHAPS at 4°C. Subsequently, 6.5 M urea and 2 M thiourea were added, and stirring was continued for 30 min at room temperature until urea and thiourea were completely dissovled. The protein extract was supplemented with 0.1 parts v/w dithiothreitol (Biorad, Munich, Germany), 0.1 parts v/w of ampholyte mixture Servalyte pH 2–4 (Serva, Heidelberg, Germany), corrected by the amount of urea added (correction factor = sample weight prior to addition of urea/sample weight after addition of urea), and stored at −80°C.

### 2-DE electrophoresis and protein identification by mass spectrometry (MS)

Liver proteins were separated by large-gel 2-DE as described previously [44]–[46]. The gel format was 40 cm (isoelectric focussing) ×30 cm (SDS-PAGE) ×0.75 mm. For isoelectric focusing (IEF) using the mobile ampholyte technique, we applied 6 µl (∼20 µg/µl) protein extract of each sample to the anodic end of an IEF-gel and used a carrier ampholyte mixture to establish a pH gradient in a range from 3 to 10. Proteins were visualized in SDS-PAGE polyacrylamide gels by high sensitivity silver staining [44].

2-DE gels were evaluated visually by a trained observer on a light box (Biotec-Fischer, Reiskirchen, Germany). Spot changes were considered with respect to quantitative variation which was quantitatively further evaluated by using the Proteomeweaver 2.1 imaging software. Protein spots found to be reproducibly altered in protein patterns of irradiated mice when compared to unirradiated heterozygous mice or to irradiated heterozygous mice were subject of further identification by mass spectrometry.

For protein identification by MS, 40 µl (∼20 µg/µl) protein extract was separated on 1.5 mm diameter IEF and 1.0 mm SDS-PAGE gels and stained with an MS-compatible silver staining protocol [47]. Protein spots of interest were excised from 2-DE gels and subjected to in-gel trypsin digestion without reduction or alkylation. Tryptic Fragments were analyzed by matrix-assisted laser desorption ionization - time of flight (MALDI-TOF) MS on a Reflex IV (Bruker Daltonics, Bremen, Germany) or nano high-performance liquide chromatography (nanoHPLC; Dionex/LC Packings, Amsterdam, Netherlands)/electrospray ionization (ESI) ion trap MS on a LCQ Deca XP (Thermo Fisher Scientific, Waltham, MA, USA). Mass spectra were analyzed using Mascot software 2.0 with automatic searches in SWISS-PROT and NCBI nonredundant databases restricted to taxonomy *Mus musculus*. Search parameters allowed for one miscleavage and for oxidation of methionine and propionamidation of cysteine. Criteria for positive identification of proteins with MS were set according to the scoring algorithm delineated in Mascot (www.matrixscience.com). Proteins were assigned to ontological groups using the ProfCom database [48]. Statistical significance of observed enrichments were determined using the two tailed Chi squared test with one degree of freedom.

### Quantification of intracellular reactive oxygen species in mouse fibroblasts

ROS measurements were made using our previously published conditional *NBN* null mutant cells [8] which were cultivated under standard conditions. Fibroblasts (5×10^5^) were treated as previously descibed [8] with a Cre recombinase fusion protein [23] 48 hours before incubation in the radiomimetic Bleomycin (30 µg/ml) to induce DNA double-strand breaks or in 0.5 mM hydrogen peroxide as a control. Cells were cultivated for 12 hours and then harvested with trypsin and resuspended in 10 µM 5-(and-6)-chloromethyl-2′,7′-dichlorodihydrofluorescein diacetate (CM-H_2_DCFDA; Invitrogen) in PBS. Incubation was for 30 minutes in the dark at 37°C. Cells were then washed and resuspended in PBS and analysed by flow cytometry on a FACS-Calibur (Becton Dickinson Biosciences) counting a minimum of 10^4^ cells per sample. Data were analysed using the WinMDI V2.9 software.

### Quantification of NAD^+^ levels in mouse liver tissue

For each animal analyzed, a piece of frozen liver tissue was ground to fine powder in a mortar precooled in liquid nitrogen. Approximately 40 mg of the tissue powder were transferred into a 1.5 ml tube, resuspended in 800 µl NAD extraction buffer (BioVision) and sonified using a model 450 sonifier (Branson Ultrasonics Corp., Danbury, CT). This was followed by filtering the samples through a 10 Kd spin column (Biovision) to remove NADH consuming enzymes. The quantification of NAD^+^ was performed using the BioVision NAD+/NADH Quantification Kit (BioVision, Mountain View, CA, USA) according to the manufacturers instructions.

## Supporting Information

Table S1 Liver protein alterations in mice heterozygous and homozygous for null mutations in the *Nbn* gene at different time points following IR.
(0.37 MB DOC)

Figure S1 Efficiency of the in vivo deletion of *Nbn* exon 6 by Cre recombinase in mouse liver tissue. The in vivo deletion efficiency of the *Nbn* exon 6 induced by Cre recombinase was assayed by analysis of immunoprecipitates from liver tissue for nibrin expression. Samples from mice of each genotype (*Nbn* ^+/del-6^ and *Nbn* ^ins-6/del-6^) and at each timepoint after IR were analysed using anti-nibrin and anti-actin antibodies for IP (Demuth I et al. 2004, Hum Mol Genet 13: 2385–2397) and for western blot (abcam and R&D Systems).
(1.77 MB TIF)

Figure S2 Protein spot alterations in livers from *Nbn* ^+/del-6^ and *Nbn* ^ins-6/del-6^ mice at different time points after irradiation with 4 Gy: Oxidative stress response proteins, metabolic proteins and heat shock proteins/chaperones. The identity of protein spots altered in protein expression was determined by mass spectrometry. Identified proteins were assigned to ontological groups by the ProfCom program. The data for three groups in which proteins were significantly enriched are shown. *Nbn* ^+/del-6^ mice in white bars, *Nbn* ^ins-6/del-6^ mice in grey bars.
(4.78 MB TIF)
