# Correction: A Systematic Proteomic Study of Irradiated DNA Repair Deficient *Nbn*-Mice

**DOI:** 10.1371/annotation/06306df9-1db3-4e7b-a7ce-18338b655967

**Published:** 2009-05-15

**Authors:** Anna Melchers, Lars Stöckl, Janina Radszewski, Marco Anders, Harald Krenzlin, Candy Kalischke, Regina Scholz, Andreas Jordan, Grit Nebrich, Joachim Klose, Karl Sperling, Martin Digweed, Ilja Demuth

The author affiliations numbers 1 and 2 are incorrect. The correct affiliations are: 1 Institut für Humangenetik, Charité - Universitätsmedizin Berlin, Campus Virchow Klinikum, Berlin, Germany, 2 Medizinische Klinik mit Schwerpunkt Hämatologie/Onkologie, Charité - Universitätsmedizin Berlin, Campus Virchow Klinikum, Berlin, Germany.

