# Supplementary material for: A Systematic Proteomic Study of Irradiated DNA Repair Deficient Nbn-Mice
Source: PLoS One. 2009 May 1;4(5):e5423. doi: 10.1371/journal.pone.0005423 (PMC2672167; doi:10.1371/journal.pone.0005423)
Supplement: Table S1 — Liver protein alterations in mice heterozygous and homozygous for null mutations in the Nbn gene at different time points following IR. (0.37 MB DOC) [file pone.0005423.s001.doc]

**Supporting Table 1**

Liver protein alterations in mice heterozygous and homozygous for null mutations in the *Nbn* gene at different time points following IR.

|  |  | ***Nbn*^+/del-6^unirradiated vs.**  ***Nbn*^+/del-6^ irradiated** | | | ***Nbn*^+/del-6^ irradiated vs.**  ***Nbn*^ins-6/del-6^ irradiated** | | |
| --- | --- | --- | --- | --- | --- | --- | --- |
| **Accession number** | **Protein**  **Time post IR** | **30min** | **2h** | **24h** | **30min** | **2h** | **24h** |
| ***A. Proteins involved in oxidative stress (oxidoreductase activity, cell redox homeostasis)*** | | | | | | | |
| O08709 | Peroxiredoxin 6 (676) |  |  |  | **▲** (1.38) |  | **▲** (1.33) |
| NP_031479 | peroxiredoxin 6 (846) | **▲ (**1**.**73**)** | **▲ (**1**.**59**)** | **▼** (1.52) | **▲ (**2**.**48**)** |  | **▲ (**2**.**16**)** |
| NP_031479 | peroxiredoxin 6 (685), |  |  |  | **▲ (**1**.**97**)** |  | **▲ (**1**.**65**)** |
| CAA28645 | manganese superoxide dismutase (144) |  |  |  |  |  | **▲ (**1**.**96**)** |
| NP_663493 | NADH dehydrogenase (921) |  | **▲ (**1**.**36**)** |  |  |  |  |
| NP_663493 | NADH dehydrogenase (1411) |  | **▼ (**2.50**)** |  |  | **▲ (**3**.**26**)** |  |
| NP_079634 | NADH dehydrogenase (ubiquinone) 1 alpha subcomplex, 9 (494) |  |  |  |  | **▲ (**1**.**79**)** | **▼ (**2.22**)** |
| BAB18776 | NADPH-dependent retinol dehydrogenase/reductase (556) |  |  |  |  |  | **▼ (**1.30**)** |
| NP_080175 | ubiquinol cytochrome c reductase core protein 2 (1058) |  |  |  |  | **▲ (**1**.**62**)** | **▲ (**1**.**31**)** |
| P19536 | cytochrome c oxidase (212), | **▲ (**8**.**40**)** | **▲ (**1**.**42**)** | **▲ (**3**.**05**)** |  |  |  |
| Q9CZ13 | ubiquinol-cytochrome c reductase core protein 1 (727) |  |  |  |  | **▲ (**1**.**48**)** | **▲ (**2**.**38**)** |
| NP_031978 | protein disulfide isomerase associated 3 (439) |  |  |  |  | **▲** (1.17) | **▼** (2.04) |
| NP_031978 | Glucose regulated protein p58 [Mus musculus] (541) |  |  |  |  |  | **▲ (**2**.**87**)** |
| NP_031978 | protein disulfide isomerase associated 3 (237) |  |  |  | **▲** (4.27) | **▲** (1.93) | **▲** (8.37) |
| NP_031978 | protein disulfide isomerase associated 3 (716) |  |  |  |  | **▼** (1.59) |  |
| P09103 | Protein disulfide isomerase precursor (122) |  |  |  | **▲ (**2**.**70**)** | **▲ (**4**.**30**)** | **▲ (**11**.**4**)** |
| NP_082235 | Protein disulfide isomerase associated 6 (975) |  |  |  |  | **▲ (**2**.**70**)** |  |
| S41661 | protein disulfide-isomerase (112) |  |  |  | **▲ (**1**.**84**)** | **▲ (**1**.**44**)** | **▲ (**5**.**04**)** |
| NP_525028 | glyoxylate reductase/hydroxypyruvate reductase (359) |  |  |  |  |  | **▼ (**6.67**)** |
| Q64105 | Sepiapterin reductase (466) |  |  |  |  |  | **▲ (**5.77**)** |
| NP_613066 | Aldh2 protein (580) |  |  |  | **▲ (**6.37**)** | **▲ (**1.55**)** | **▲ (**20.2**)** |
| NP_064377 | aldehyde dehydrogenase 9 (1124) |  |  |  |  | **▼ (**3.85**)** | **▼ (**20.0**)** |
| NP_032318 | hydroxysteroid (17-beta) dehydrogenase (481) |  |  |  |  |  | **▲ (**1.62**)** |
| AAH31710 | RIKEN cDNA 1300018L09 gene (712) |  |  |  |  | **▼ (**1.92**)** | **▼ (**1.47**)** |
| NP_032318 | hydroxysteroid (17-beta) dehydrogenase 4 (270) |  | **▼ (**1.89**)** |  |  |  | **▲ (**1**.**42**)** |
| NP_079610 | RIKEN cDNA 0610040B21 (342) |  |  |  | **▲ (**2**.**20**)** |  | **▲ (**3**.**54**)** |
| NP_032644 | Malate dehydrogenase (217) |  |  |  | **▼ (**1.54**)** |  | **▲ (**2**.**71**)** |
| NP_083849 | isocitrate dehydrogenase 3 (NAD+) alpha (168) |  |  |  | **▲ (**1.39**)** | **▲ (**2.59**)** | **▲ (**1.56**)** |
| NP_083849 | isocitrate dehydrogenase 3 (223) |  |  |  |  |  | **▼ (**1.92**)** |
| NP_075629 | glutaredoxin 3 (604) |  |  |  |  |  |  |
| AAH27270 | Ndufs3 protein (445) | **▼ (**2.86**)** | **▲ (**1**.**56**)** | **▼ (**2.56**)** |  |  | **▼ (**1.79**)** |
| NP_034404 | glycerol-3-phosphate dehydrogenase 2 (1124) |  |  |  |  |  | **▼ (**3.57**)** |
| NP_619606 | sarcosine dehydrogenase (152) |  |  |  |  |  | **▲ (**49**.**1**)** |
| NP_758468 | choline dehydrogenase (588) |  |  |  |  |  | **▲ (**1**.**35**)** |
| NP_033500 | urate oxidase; uricase (562) |  |  |  | **▲ (**1**.**84**)** |  |  |
| NP_613066 | aldehyde dehydrogenase family 7 (1024) |  |  |  |  |  | **▼** (2.33) |
| NP_613066 | aldehyde dehydrogenase family 7 (910) |  |  |  |  |  | **▼ (**1.33**)** |
| AAB37274 | aldo-keto reductase family 1 (267) |  |  |  |  |  | **▲ (**1**.**29**)** |
| NP_083831 | class kappa glutathione S-transferase* (142) |  |  |  |  | ▲ (2.60) | ▼ (2.56) |
| NP_032206 | glutathione synthetase* (1237) |  |  | ▲ (3.59) | ▲ (1.55) | ▲ (1.45) | ▲ (4.04) |
| ***B. Proteins involved in metabolism (metabolic process)*** | | | | | | | |
| NP_032823 | pyruvate carboxylase (157) |  |  |  | **▲ (**2.20**)** |  | **▲ (**2.56**)** |
| NP_032823 | pyruvate carboxylase (175) |  |  |  |  |  | **▲ (**1.34**)** |
| NP_032823 | pyruvate carboxylase (530) |  |  |  |  | **▲ (**1.31**)** | **▲ (**1.42**)** |
| NP_032823 | pyrovate carboxylase (887) |  |  |  |  |  | **▲ (**1.21**)** |
| NP_032823 | Pcx protein (=pyrovate carboxylase) (637) |  |  |  |  |  | **▲ (**1.55**)** |
| NP_525028 | glyoxylate reductase/hydroxypyruvate reductase (359) |  |  |  |  |  | **▼** (6.67) |
| NP_543121 | formiminotransferase cyclodeaminase (72) |  |  |  |  |  | **▲ (**1.46**)** |
| Q64105 | Sepiapterin reductase (466) |  |  |  |  |  | **▲ (**5.77**)** |
| AAH54425 | Succinyl-CoA ligase [GDP-forming] beta-chain (996) |  |  |  |  |  | **▼** (1.59) |
| NP_058052 | enoyl coenzyme A hydratase 1 (506) |  |  |  | **▲ (**1.49**)** | **▲ (**1.81**)** |  |
| NP_084501 | dihydrolipoamide S-succinyltransferase (208), |  |  |  |  |  | ▲ (2.28) |
| NP_001074278 | similar to Carbamoyl-phosphate synthase (122) |  | **▲** (2.14) |  |  |  | **▲** (1.36) |
| NP_001074278 | Carbamoyl-phosphate synthase 1 (496) |  |  |  |  |  | **▲ (**2.16**)** |
| NP_001074278 | similar to Carbamoyl-phosphate synthase (1224) |  |  |  | **▲ (**1.61**)** |  | **▲ (**2.13**)** |
| XP_129769 | similar to Carbamoyl-phosphate synthase (101) |  |  | **▲** (5.66) |  |  | **▼** (1.92) |
| NP_001074278 | similar to Carbamoyl-phosphate synthase (541) |  |  |  |  |  | **▲ (**1.34**)** |
| XP_129769 | similar to Carbamoyl-phosphate synthase (614) |  |  |  |  |  | **▲ (**1.24**)** |
| AAH55890 | dihydrolipoamide branched chain transacylase E2 (107) |  |  |  | **▲** (1.22) | **▲** (1.35) | **▲** (3.19) |
| NP_613066 | Aldh2 protein (580) |  |  |  | **▲ (**6.37**)** | **▲ (**1.55**)** | **▲ (**20.2**)** |
| NP_064377 | aldehyde dehydrogenase 9 (1124) |  |  |  |  | **▼ (**3.85**)** | **▼ (**20.0**)** |
| NP_659152 | aldolase 2, B isoform (504) |  |  |  |  |  | **▲ (**1.53**)** |
| NP_035658 | Transaldolase 1 (615) |  |  |  |  |  | **▼ (**6.25**)** |
| NP_035658 | Transaldolase 1 (436) |  |  |  |  |  | **▼ (**2.94**)** |
| NP_035658 | transaldolase 1 (237) |  |  |  |  |  | ▼ (3.85) |
| 1GLP_A | Chain A, Glutathione S-Transferase Yfyf (Class Pi) (452) |  |  |  | **▼ (**1.47**)** | **▲ (**3.83**)** | **▲ (**8.01**)** |
| P10648 | glutathione transferase (99) |  | **▼ (**1.45**)** |  |  |  |  |
| NP_032318 | hydroxysteroid (17-beta) dehydrogenase (481) |  |  |  |  |  | **▲ (**1.62**)** |
| AAH31710 | RIKEN cDNA 1300018L09 gene (712) |  |  |  |  | **▼ (**1.92**)** | **▼ (**1.47**)** |
| NP_083849 | isocitrate dehydrogenase 3 (NAD+) alpha (168) |  |  |  | **▲ (**1.39**)** | **▲ (**2.59**)** | **▲ (**1.56**)** |
| NP_083849 | isocitrate dehydrogenase 3 (223) |  |  |  |  |  | **▼ (**1.92**)** |
| BAB18776 | NADPH-dependent retinol dehydrogenase/reductase (556) |  |  |  |  |  | **▼ (**1.30**)** |
| ***C. Chaperones and heat shock proteins (unfolded protein binding)*** | | | | | | | |
| AAB21806 | heat shock protein hsp60 (608) |  |  |  | **▲ (**1.82**)** |  | **▲ (**3.79**)** |
| AAB21806 | heat shock protein hsp60 (234) |  |  |  | **▲ (**1.75**)** |  | **▲ (**4.56**)** |
| AAB21806 | heat shock protein hsp60 (352) |  |  |  |  | **▲ (**3.18**)** | **▲ (**10.9**)** |
| AAB21806 | heat shock protein 1 (851) |  |  |  |  | **▲ (**2.01**)** |  |
| AAB21806 | heat shock protein hsp60 (46), |  |  |  |  | **▲ (**2.46**)** | **▲ (**2.31**)** |
| AAB21806 | heat shock protein hsp60 (222) |  |  |  | **▲ (**1.58**)** | **▲ (**1.83**)** | **▲ (**2.07**)** |
| AAB21806 | heat shock protein hsp60 (438) |  |  |  |  |  | **▲ (**1.43**)** |
| AAB21806 | HSP60 (1146) |  |  |  |  |  | **▲** (1.60) |
| NP_032324 | DnaJ-like protein 2=HSP40 (692) |  |  |  |  |  | **▲ (**2.38**)** |
| NP_080784 | TNF receptor-associated protein 1 (TRAP-1)(1958) |  |  |  |  | **▼ (**1.28**)** | **▼ (**4.17**)** |
| NP_080784 | TNF receptor-associated protein 1 (TRAP-1) (86) |  |  |  |  |  | **▼ (**1.64**)** |
| NP_062768 | DnaJ (Hsp40) homolog (106) |  |  |  | **▲ (**1.28**)** |  |  |
| NP_031662 | chaperonin subunit 2 (beta) (1938) |  |  |  |  | **▼** (1.82) | **▼** (5.88) |
| NP_035761 | tumor rejection antigen gp96 (1016) |  |  |  |  | **▲ (**1.55**)** |  |
| ***D. Proteins involved in cell cycle regulation*** | | | | | | | |
| NP_009057 | valosin-containing protein (125) |  |  |  | **▲ (**1**.**30**)** |  | **▲ (**4**.**30**)** |
| NP_077168 | SEC13 related gene (364) |  | **▼** (2.44**)** |  |  |  |  |
| AAA37667 | beta-galactoside binding protein (270) | **▼** (2.00) | **▼** (3.03) | **▼ (**2.86**)** |  |  |  |
| NP_080750 | SGT1 (282) |  |  |  |  | **▲ (**1**.**97**)** | **▲ (**4**.**96**)** |
| NP_036131 | COP9 signalosome subunit 4 (738) |  |  |  |  |  |  |
| NP_038692 | SH domain Grb2 like (89) |  |  |  |  |  | **▲ (**1**.**62**)** |
| NP_035249 | proliferation-associated 2G4 (137) |  |  |  |  |  | **▼ (**1.75**)** |
| NP_031501 | acidic ribosomal phosphoprotein P0 (928) |  |  |  |  |  | **▼ (**2.63**)** |
| ***E. Proteasomal proteins*** | | | | | | | |
| AAH37127 | Atp5b, proteasome subunit alpha typ 3 (469) |  |  |  |  |  | **▲** (2.24) |
| O70435 | Proteasome subunit alpha type 3 (581) |  |  |  |  | **▼ (**1.82**)** |  |
| O70435 | Proteasome subunit alpha type 3 (442) |  |  |  |  | **▲ (**4**.**09**)** |  |
| NP_032973 | protease (prosome, macropain) 26S subunit (1075) |  |  |  |  |  | **▼ (**1.92**)** |
| NP_005796 | 26S proteasome-associated pad1 homolog (59) |  |  |  | **▲ (**1**.**76**)** | **▲ (**1**.**22**)** | **▲ (**2**.**53**)** |
| NP_035317 | proteasome (prosome, macropain) subunit, beta type 7 (366) |  |  |  |  | **▼ (**1.35**)** | **▼ (**1.27**)** |
| ***F. Proteins involved in nucleic acids metabolism*** | | | | | | | |
| P23492 | Purine nucleoside phosphorylase (841) |  |  |  |  |  | **▼ (**1.37**)** |
| AAH11036 | bisphosphate 1,3 nucleotidase (554) |  |  |  | **▲ (**1**.**66**)** | **▲ (**1**.**66**)** | **▼ (**2.13**)** |
| AAD17330 | bisphosphate 3'-nucleotidase (464) |  | **▲ (**5**.**62**)** |  |  | **▼ (**1.56**)** |  |
| AAD17330 | bisphosphate 1,3 nucleotidase (168) |  |  |  |  | **▲ (**3**.**85**)** | **▲ (**1**.**42**)** |
| ***G. Proteins involved in gene expression*** | | | | | | | |
| NP_084385 | splicing factor 3b, subunit 2 [Mus musculus] (113) |  |  |  |  |  | **▲ (**3**.**20**)** |
| NP_032313 | Heat Responsive Protein 12 (191) |  |  |  |  |  | **▲ (**5**.**08**)** |
| O88569 | heterogeneous nuclear ribonucleoprotein A2/B1/B0 (406) |  |  |  |  |  | **▲ (**1**.**36**)** |
| ***H. Proteins involved in apoptosis*** | | | | | | | |
| NP_076360 | SET translocation (572) |  |  |  |  | **▼ (**1.59**)** | **▲ (**2**.**39**)** |
| NP_080270 | EF hand domain containing 2 (601) |  | **▼** (1.23) |  |  |  |  |
| ***I. Protease inhibitors*** | | | | | | | |
| NP_033273 | serine (or cysteine) proteinase inhibitor (303) |  |  |  | **▲** (1.30) |  |  |
| AAH11217 | serine protease inhibitor A3K (739) |  |  | **▼ (**1.20**)** |  |  |  |
| ***K. other proteins*** | | | | | | | |
| NP_084090 | chromatin modifying protein 5 (164) |  |  |  |  | **▲ (**2**.**16**)** |  |
| XP_484006 | hypothetical protein (178) |  |  | **▼** (1.45) |  | **▼** (3.23) |  |
| NP_081147 | histidine triad protein 3 (142) |  | **▲ (**3**.**48**)** |  |  |  |  |
| O55023 | Inositol-1(or 4)-monophosphatase (390) |  |  |  |  | **▲** (1.62) | **▲** (2.93) |
| NP_001605 | cytoskeletal gamma-actin (178) |  |  |  | **▲** (1.85) |  | **▲** (2.10) |
| NP_058659 | Tropomodulin 3 (239) |  |  | **▲ (**3**.**94**)** | **▲ (**3**.**34**)** | **▲ (**7**.**75**)** | **▲ (**5**.**95**)** |
| CAA43087 | radixin (907) |  |  |  |  |  | **▼ (**6.25**)** |
| CAA31455 | gamma-actin (274) |  |  |  | **▲ (**2**.**94**)** | **▲ (**3**.**48**)** | **▲ (**6**.**01**)** |
| BAA33770 | heme-binding protein (355) |  |  |  | ▲ (1.27) | ▲ (3.11) | ▲ (2.04) |
| NP_058972 | guanosine diphosphate (GDP) dissociation inhibitor 3 (897) |  |  |  |  |  | **▼** (1.59) |
| P17563 | Major Urinary Protein Complex (699) |  |  |  |  | **▲ (**7**.**27**)** | **▲ (**1**.**30**)** |
| NP_112465 | Selenium binding protein 1 (124) |  |  |  |  | **▲ (**2**.**64**)** | **▲ (**3**.**13**)** |
| NP_062287 | Selenium binding protein 2 (422) |  |  |  |  |  | **▲ (**4.63**)** |
| NP_062287 | Selenium binding protein 2 (1070) |  |  |  | **▲ (**2**.**34**)** |  | **▲ (**3**.**84**)** |
| NP_033176 | selenium binding protein 1(346) | **▲ (**2**.**02**)** | **▲ (**3**.**01**)** | **▲ (**1**.**43**)** |  |  |  |
| NP_033176 | selenium binding protein 1 (750) |  |  |  |  |  | **▲ (**2**.**26**)** |
| NP_062287 | selenium binding protein 2 (707) |  |  |  |  | **▲ (**1**.**93**)** | **▲ (**4**.**70**)** |
| NP_062287 | selenium binding protein 2 (609) | **▲ (**2**.**08**)** |  |  |  |  | **▲ (**2**.**69**)** |
| NP_034157 | D-dopachrome tautomerase (285) |  |  |  |  |  | **▼ (**1.39**)** |
| AAH21949 | 2810413N20Rik protein (110) |  |  |  |  | **▲ (**8**.**01**)** |  |
| AAH30849 | AW124722 protein (551) |  |  |  |  | **▼** (3.57) |  |
| AAH24619 | 6430559E15Rik protein (725) |  | **▼ (**10.0**)** | **▼ (**3.33**)** | **▲ (**2**.**82**)** |  |  |
| NP_083907 | RIKEN cDNA 1810013B01 (484) |  | **▲ (**1**.**74**)** | **▲ (**1**.**73**)** |  | **▼ (**1.35**)** | **▼ (**1.59**)** |
| BAC36005 | unnamed protein product (1205) |  | **▲ (**1**.**88**)** | **▲ (**1**.**59**)** |  | **▼ (**4.17**)** |  |
| BAC26064 | unnamed protein product (459) |  |  |  |  |  | **▼ (**1.75**)** |
| AAH04005 | Elongation factor 1-alpha 1 (EF-1-alpha-1) (134) |  |  |  |  |  | **▲ (**1**.**54**)** |
| NP_031531 | ATP synthase, H+ transporting, mitochondrial F1 complex, alpha subunit (236) |  |  |  |  |  | ▲ (3.87) |
| NP_031531 | ATP synthase, H+ transporting, mitochondrial F1 complex, alpha subunit (122) |  |  |  |  | ▼ (1.47) | ▼ (2.04) |
| AAB86241 | ATP synthase beta-subunit (230) |  |  |  | ▲ (2.83) | ▲ (3.07) | ▲ (3.60) |
| P97328 | Ketohexokinase (83) |  |  |  |  | ▲ (3.86) | ▲ (4.26) |
| NP_034385 | guanidinoacetate methyltransferase (119) |  |  |  | ▲ (1.40) |  | ▲ (5.14) |
| AAH04017 | enolase 1 (527) |  |  |  |  |  | ▲ (2.17) |
| AAH04017 | enolase 1 (230) |  |  |  | ▲ (1.35) | ▲ (6.64) | ▲ (2.19) |
| NP_031632 | carbonic anhydrase 3 (498) |  | ▼ (1.54) | ▼ (2.13) |  |  | ▲ (1.22) |
| NP_065266 | carnitine/acylcarnitine translocase (457) |  | ▼ (1.35) | ▲ (1.92) |  |  |  |
| Q5FW57 | Glycine N-acyltransferase-like protein (407) |  |  |  |  |  |  |
| NP_057877 | betaine-homocysteine methyltransferase (392) |  |  |  | ▲ (1.23) |  |  |
| NP_058674 | ornithine aminotransferase (594) |  |  |  |  |  | ▲ (1.78) |
| NP_058674 | ornithine aminotransferase (325) |  |  |  |  |  | ▲ (4.11) |
| NP_058674 | ornithine aminotransferase (1332) |  |  |  |  | ▼ (2.00) | ▼ (1.67) |
| NP_079647 | aminoacylase 1 (436) |  |  |  | ▼ (2.13) |  |  |
| NP_031520 | Argininosuccinate synthetase (472) |  |  |  | ▼ (1.23) | ▲ (1.91) | ▼ (1.19) |
| NP_034455 | glutamate oxaloacetate transaminase 2, mitochondrial (672) |  | ▲ (2.24) | ▲ (2.42) |  |  |  |
| NP_666065 | cystathionase; cystathionine gamma-lyase (1051). |  |  |  |  |  | ▲ (1.34) |
| NP_080405 | Endoplasmic reticulum protein ERp29 precursor (301) |  | ▲ (2.72) | ▲ (5.64) |  |  | ▼ (10.0) |

For each altered protein the SWISS-PROT/TrEMBL or NCBI accession number is given together with the Mowse score, in brackets, calculated by the MASCOT software. Upward and downward triangles indicate an increase or decrease of protein spot intensity, respectively, with the median fold increase or decrease of spot intensity given in brackets.

^*^Not included in the list of proteins involved in oxidative stress by ProfCom results but independently added based on strong evidence in the literature [1,2] (review)

**References**

1. Jowsey IR, Thomson RE, Orton TC, Elcombe CR, Hayes JD (2003) Biochemical and genetic characterization of a murine class Kappa glutathione S-transferase. Biochem J 373: 559-569.

2. Njalsson R, Norgren S (2005) Physiological and pathological aspects of GSH metabolism. Acta Paediatr 94: 132-137.
