# Supplementary figures and images for: A Systematic Proteomic Study of Irradiated DNA Repair Deficient Nbn-Mice
Source: PLoS One. 2009 May 1;4(5):e5423. doi: 10.1371/journal.pone.0005423 (PMC2672167; doi:10.1371/journal.pone.0005423)

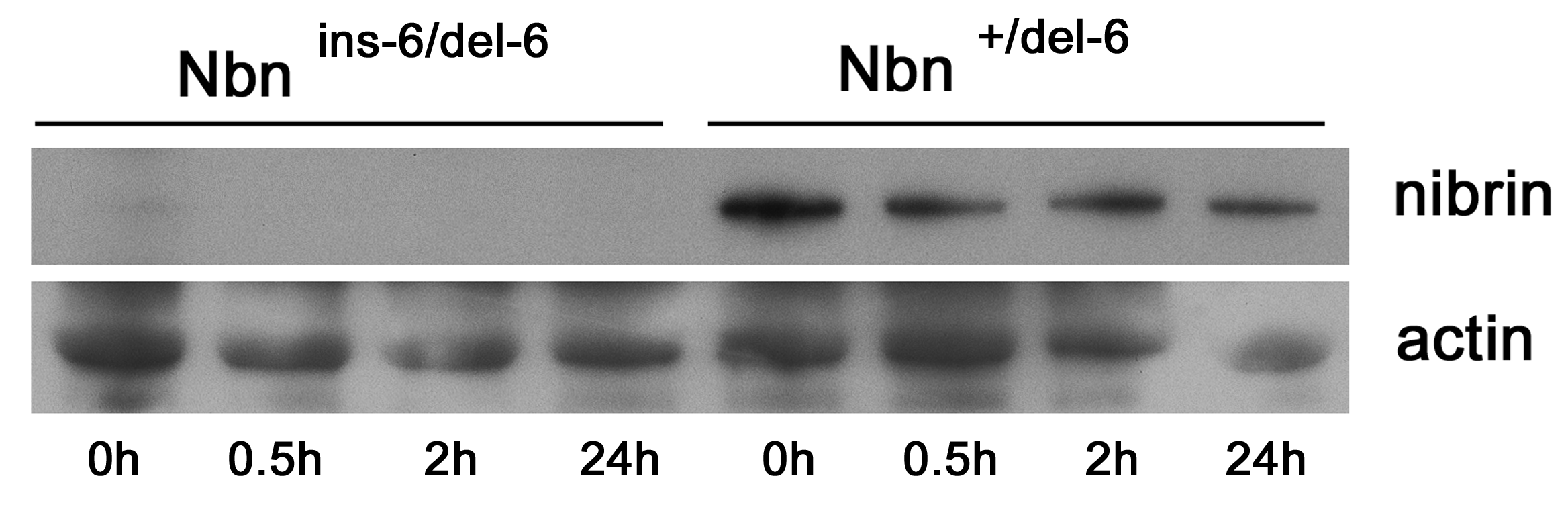

Supplement: Figure S1 — Efficiency of the in vivo deletion of Nbn exon 6 by Cre recombinase in mouse liver tissue. The in vivo deletion efficiency of the Nbn exon 6 induced by Cre recombinase was assayed by analysis of immunoprecipitates from liver tissue for nibrin expression. Samples from mice of each genotype (Nbn +/del-6 and Nbn ins-6/del-6) and at each timepoint after IR were analysed using anti-nibrin and anti-actin antibodies for IP (Demuth I et al. 2004, Hum Mol Genet 13: 2385–2397) and for western blot (abcam and R&D Systems). (1.77 MB TIF) [file pone.0005423.s002.tif]

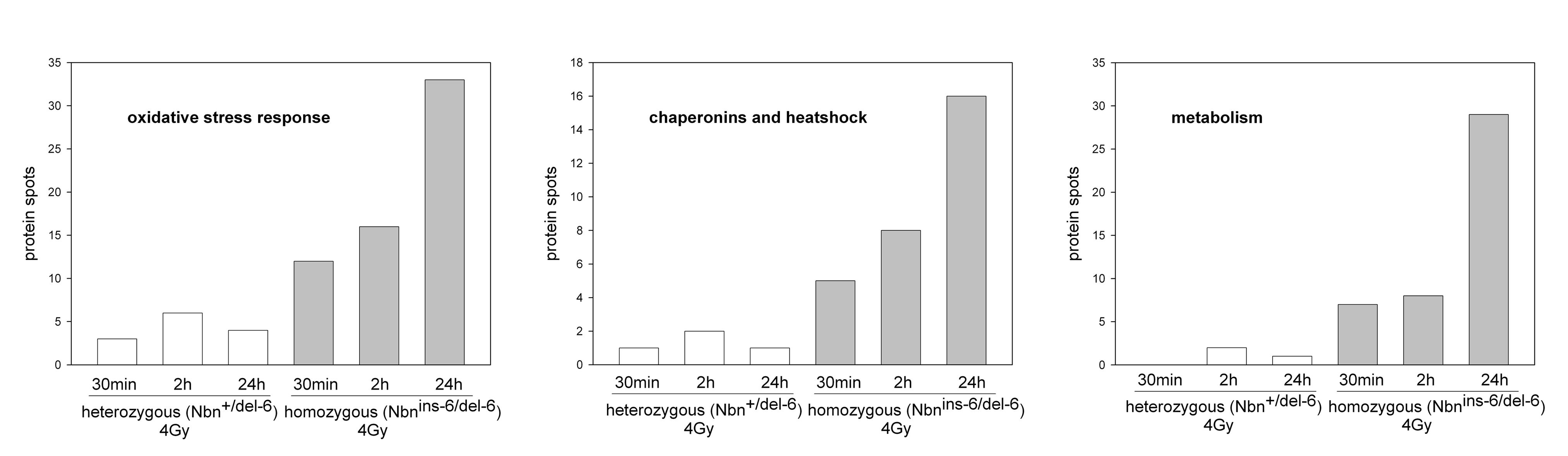

Supplement: Figure S2 — Protein spot alterations in livers from Nbn +/del-6 and Nbn ins-6/del-6 mice at different time points after irradiation with 4 Gy: Oxidative stress response proteins, metabolic proteins and heat shock proteins/chaperones. The identity of protein spots altered in protein expression was determined by mass spectrometry. Identified proteins were assigned to ontological groups by the ProfCom program. The data for three groups in which proteins were significantly enriched are shown. Nbn +/del-6 mice in white bars, Nbn ins-6/del-6 mice in grey bars. (4.78 MB TIF) [file pone.0005423.s003.tif]
